# Amyloid-Beta Induced CA1 Pyramidal Cell Loss in Young Adult Rats Is Alleviated by Systemic Treatment with FGL, a Neural Cell Adhesion Molecule-Derived Mimetic Peptide

**DOI:** 10.1371/journal.pone.0071479

**Published:** 2013-08-09

**Authors:** Nicola J. Corbett, Paul L. Gabbott, Boris Klementiev, Heather A. Davies, Frances M. Colyer, Tatiana Novikova, Michael G. Stewart

**Affiliations:** 1 The Open University, Department of Life, Health and Chemical Sciences, Milton Keynes, United Kingdom; 2 University of Copenhagen, Department of Neuroscience and Pharmacology, Copenhagen, Denmark; Federal University of Rio de Janeiro, Brazil

## Abstract

Increased levels of neurotoxic amyloid-beta in the brain are a prominent feature of Alzheimer’s disease. FG-Loop (FGL), a neural cell adhesion molecule-derived peptide that corresponds to its second fibronectin type III module, has been shown to provide neuroprotection against a range of cellular insults. In the present study impairments in social recognition memory were seen 24 days after a 5 mg/15 µl amyloid-beta(_25–35_) injection into the right lateral ventricle of the young adult rat brain. This impairment was prevented if the animal was given a systemic treatment of FGL. Unbiased stereology was used to investigate the ability of FGL to alleviate the deleterious effects on CA1 pyramidal cells of the amyloid-beta(_25–35_) injection. NeuN, a neuronal marker (for nuclear staining) was used to identify pyramidal cells, and immunocytochemistry was also used to identify inactive glycogen synthase kinase 3beta (GSK3β) and to determine the effects of amyloid-beta(_25–35_) and FGL on the activation state of GSK3β, since active GSK3β has been shown to cause a range of AD pathologies. The cognitive deficits were not due to hippocampal atrophy as volume estimations of the entire hippocampus and its regions showed no significant loss, but amyloid-beta caused a 40% loss of pyramidal cells in the dorsal CA1 which was alleviated partially by FGL. However, FGL treatment without amyloid-beta was also found to cause a 40% decrease in CA1 pyramidal cells. The action of FGL may be due to inactivation of GSK3β, as an increased proportion of CA1 pyramidal neurons contained inactive GSK3β after FGL treatment. These data suggest that FGL, although potentially disruptive in non-pathological conditions, can be neuroprotective in disease-like conditions.

## Introduction

Alzheimer’s disease (AD) pathology includes formation of amyloid plaques and neurofibrillary tangles, neuroinflammation [1], neurotransmitter deficits [2], synaptic alterations [3] and neuronal cell loss [4]. A decrease has been noted in the density and total number of neurons in the temporal cortex, frontal cortex [5]–[7], entorhinal cortex, particularly layers II and IV [8], [9], the Nucleus Basalis of Meynert, the locus coeruleus [7], [10], cerebellum [11] and hippocampus correlating to regional atrophy in AD [12]. Mann et al. (1985a) found that in the temporal cortex there is a direct correlation between neuronal cell loss, and amyloid plaque and neurofibrillary tangle accumulation [13]. In both the temporal cortex and frontal cortex, Hansen et al. (1988) found a 15 to 18% decrease in neuronal density in late stage cases of AD but there was actually a greater neuronal loss (23 to 26% decrease) in the early stages of AD [14]. The most well known feature of AD, memory impairment (particularly episodic and spatial memory), is correlated with decreased hippocampal volume [15] due to the dysfunction of neurons and synapses in the CA1 and entorhinal cortex [16]–[19].

At present there is no effective treatment for AD; only short term means to alleviate symptoms [20]. Previous work from our group has shown that FG-Loop (FG-Loop - FGL), a neuronal cell adhesion molecule (NCAM)-derived peptide that is known to be an agonist of the fibroblast growth factor receptor (FGFR), may act as a neuroprotective agent in AD [21]. FGL mimics a 15 amino acid long segment of the second fibronectin type III homology module of the NCAM close to the N-terminal in the turn of the F and G β strands (E^681^VYVVAENQQGKSKA^695^; [22]). This site in NCAM was able to bind to the immunoglobulin-like domain D3 of the FGFR1 [22] and FGFR2 [23]. FGL has been shown to be neuroprotective in a range of pathological situations in vitro [24], and in vivo in the aged rodent [25]–[27], the ischemic male Mongolian gerbil model [28] and, of particular interest for the current study, in the cingulate cortex and CA3 of the amyloid beta_25–35_ (Aβ_25–35_-)-injected rat brain [21]. The NCAM-derived peptide has also been shown to be anti-inflammatory both in vitro and in vivo, particularly in the aged rat hippocampus [26], [29], [30], and a cognitive enhancer [28], [31], [32]. All the effects of FGL have been dependent on the activation of FGFR1 and FGFR2 rather than NCAM-induced signalling [24]. Neiiendam et al. (2004) found the activation of FGFR led to downstream activation of mitogen-activated protein/extracellular-regulated kinase kinase (MEK), phosphatidylinositol-3-kinase (PI3K), and phosphorylation of extracellular signal-regulated kinase 1/2 (ERK1/2) and protein kinase B (AKT) [24]. Klementiev et al. (2007) have shown that FGL activity was associated with an increased inhibition of glycogen synthase kinase 3β (GSK3β), which is downstream from AKT [21]. FGL may be beneficial in AD via the inhibition of GSK3β, as the activation of GSK3β, possibly via Aβ accumulation is known to cause many AD pathologies such as further Aβ accumulation, tau hyperphosphorylation and apoptosis [33].

Although transgenic animal models are particularly useful tools to study the pathology of familial AD, injections of different fragments of Aβ have provided an important experimental approach without manipulating genetic make up, when considering sporadic AD, and investigating the amyloid cascade hypothesis. One of these injection models has been used in the current study; it involves the injection of the Aβ_25–35_ fragment into the right lateral ventricle (for ease of handling due to the dexterity of the researcher) of the rat brain. Previous studies have shown that Aβ_25–35_ is equally as neurotoxic as full length Aβ_1–42_ causing cell degeneration and loss [21], [34]–[37] increased immunoreactivity of phosphorylated tau [38], learning and memory impairments [21], [39]–[41] and inflammatory upregulation [21], whilst promoting endogenous production of Aβ_1–40_ and Aβ_1–42_ [21]. Whilst FGL has been shown to alleviate the effects of Aβ little is known about its effect on neurons in the hippocampus. The aim of the present investigation was to elaborate further previous findings by Klementiev et al., 2007 [21] by carrying out a morphological examination of the effects of a single intracerebroventricular (i.c.v.) injection of Aβ_25–35_ on the young adult rat hippocampus, and following systemic treatment of FGL, specifically in the hippocampal regions, implicated in memory formation.

## Materials and Methods

### Ethical Statement

This study was performed in strict accordance with Danish legislation. An animal licence was obtained from the Danish animal experiments inspectorate (2001/561–483). Administration of Aβ_25–35_ or vehicle was performed under anaesthesia using an intraperitoneal (i.p.) injection of Hypnorm/Midazolam (23.6 µg fentanyl, 0.75 mg fluanisone, 375 µg midazolam/100 g animal; 0.3 ml/100 g, Pharmacy of the Royal Veterinary and Agricultural University, Frederiksberg, Denmark) and every effort was made to minimize suffering.

### Experimental Animals

Young adult male Wistar rats (300 g at the start of the experiment; Charles River, Sulzfeld, Germany) were housed in cages (2 per cage) with free access to food and water, in a regulated environment (23°C, 50% humidity, diurnal 12 hour light/dark cycle). The rats were split equally into 4 groups (n = 4; Aβ_25–35_+vehicle, Aβ_25–35_+FGL, vehicle+FGL, control).

### Intracerebroventricular Administration of Amyloid-beta_25–35_

Aggregates of Aβ_25–35_ (Bachem AG, Weil am Rhein, Germany) were prepared by incubating the peptide at a concentration of 3 µg/µl in distilled water for 4 days at 37°C prior to administration, as previously described by Delobette et al. (1997) to form fibril-like structures and globular amporphous aggregates [39]. Two months from the date of delivery of the animals, 5 µg/15 µl of aggregated Aβ_25–35_ or distilled water as a vehicle, were i.c.v. injected (the tip of the syringe needle being 0.8 mm posterior to bregma, 1.5 mm lateral to the sagittal suture and 3.8 mm beneath the surface of the brain) into the right lateral ventricle using a 10 µl syringe on day 0 of the experiment. These injections were administered between 10 am and 1 pm, during the light part of the cycle.

### Subcutaneous Administration of FGL_L_

FGL_L_, consisting of two FGL monomers linked via aminodiacetic acid through their N terminal, was synthesised by Polypeptide Laboratories (Hillerød, Denmark) as mentioned in Secher et al. (2006) [32] and Klementiev et al. (2007) [21]. 2 ml/kg (10.8 mg/kg) of FGL_L_ was dissolved in 0.5% w/v albumin bovine serum (BSA; Sigma-Aldrich Company LTD, Gillingham, UK) in 0.01 M phosphate saline buffer (PBS, pH 7.4). Seven days after the i.c.v. injection and every third day up to, and including, day 25 of the experiment, either FGL_L_ or 0.5% w/v BSA in 0.01 M PBS, as a vehicle, was subcutaneously (s.c.) administered using a 1 ml sterile syringe. These treatments were administered also during the light part of the cycle, between 2 pm and 3 pm without anaesthesia.

Secher et al. (2006) used an enzyme-linked immunosorbent assay (ELISA) to determine the concentration of FGL_L_ in the plasma and cerebrospinal fluid (CSF) of adult rats after s.c. administration [32]. They found that FGL_L_ was detectable in the plasma and CSF 10 minutes after administration and was still detectable up to five hours later, which suggests FGL_L_ is able to cross the blood brain barrier [32]. The hippocampus is a major target for FGL [26], with phosphorylation of FGFR1 in the hippocampus occurring within one hour of s.c. administration of the peptide [42].

### Social Recognition Memory Test

On day 24 (one day prior to the final FGL treatment), short-term memory was measured using the social recognition memory test [43]. The test rats were placed into individual cages 15 minutes before a novel juvenile male Wistar rat was introduced. The juvenile rat was left in the cage for four minutes and then removed. After a 30-minute interval, the same juvenile rat was placed in the cage. The time taken to investigate the juvenile was recorded on both occasions. Investigatory behaviours included direct contact with inspection and sniffing of the juvenile’s body, and also following the juvenile rat closely [43]. The social recognition ratio was calculated from the times spent investigating the juvenile during each encounter. If the rat had no memory of the first encounter there would be no difference between the two times and the ratio would be 0.50. Whilst the lower the ratio, compared with 0.5, the quicker the rat was at recognizing the juvenile during the second exposure.

### Transcardial Perfusion and Tissue Sectioning

On day 27 (two days after the final FGL treatment), each animal was given a terminal i.p. dose of sodium pentobarbitone (200 mg/kg; Pharmacy of the Royal Veterinary and Agricultural University, Frederiksberg, Denmark). The animals were transcardially perfused with 100 ml of 0.9% w/v sodium chloride and 0.5% w/v heparin sodium salt (from Porcine intestinal mucosa – endotoxin free; Sigma-Aldrich Company LTD, Gillingham, UK) in distilled water at a flow rate of 1.08 ml/second using a peristaltic pump (Watson Marlow Bredel Pumps digital 505 s with 313 D Pumphead 3 rollers, 1.6 mm wt tubing, Falmouth, UK). This was followed by 50 ml of fixative solution; 3.75% w/v acrolein (TAAB Laboratories Equipment LTD, Aldermaston, UK) and 2% w/v paraformaldehyde (Sigma-Aldrich Company LTD, Gillingham, UK) in 0.1 M phosphate buffer (PB, pH 7.4), at a rate of 1.63 ml/second. Finally, a perfusion of approximately 400 ml of 2% w/v paraformaldehyde in 0.1 M PB, pH 7.4 at a rate of 1.08 ml/second. Whole brains were then carefully removed from the skull. The hippocampus (−1.60 mm to −7.04 mm relative to bregma [44]) was coronally cut out of each brain as a block and serially sectioned in the coronal plane in a bath of 0.1 M PB, pH 7.4 using a Leica vT1000S vibrating microtome (Leica Microsystems LTD, Milton Keynes, UK). The majority of sections were cut at 50 µm, with every fifth section cut at 100 µm for different histological and immunocytochemical techniques. The section at which the CA3 was visible in the ventral region of the hippocampus (bregma −4.00 mm) was noted for dorsal-ventral analysis.

### Volume Estimation

The volume of the hippocampus and its subregions were calculated using the Cavalieri principle. The first 50 µm section was taken from every 1-in-5 series of sections throughout the hippocampus (an average of 21 sections per hippocampus with 300 µm between each section). The sections were stained with 0.5% w/v Toluidine blue (Agar Scientific LTD, Stansted, UK) in distilled water, mounted on gelatin-coated glass slides, dehydrated through an ascending series of alcohols (Hayman LTD, Witham, UK), passed through xylene (VWR International BDH, Lutterworth, UK) and glass cover slips were applied to the slides using Pertex (Cell path LTD, Newtown, UK). Images of the hippocampus were captured using a Nikon DXM1200 digital camera attached to a Nikon Eclipse E800 microscope (Nikon UK LTD, Kingston-upon-Thames, UK) at a magnification of 10X (Nikon Plan Fluor 1x/0.30 and optical lens CFI 10x/22). Images were then layered, aligned and stacked using Adobe Photoshop CS2 version 9.0 (Adobe Systems Europe LTD, Uxbridge, UK). The stacks were exported to the freely available reconstruction program ‘IGL Trace’ version 1.24 b [45]. The right and left hippocampi, and their cytoarchitecturally distinct subregions, as defined by Paxinos and Watson (1998) [44], and West et al. (1991) [46] were then outlined throughout the stack and the program calculated volume estimations for each structure.

### Double Immunohistochemistry

**Figure 1:**
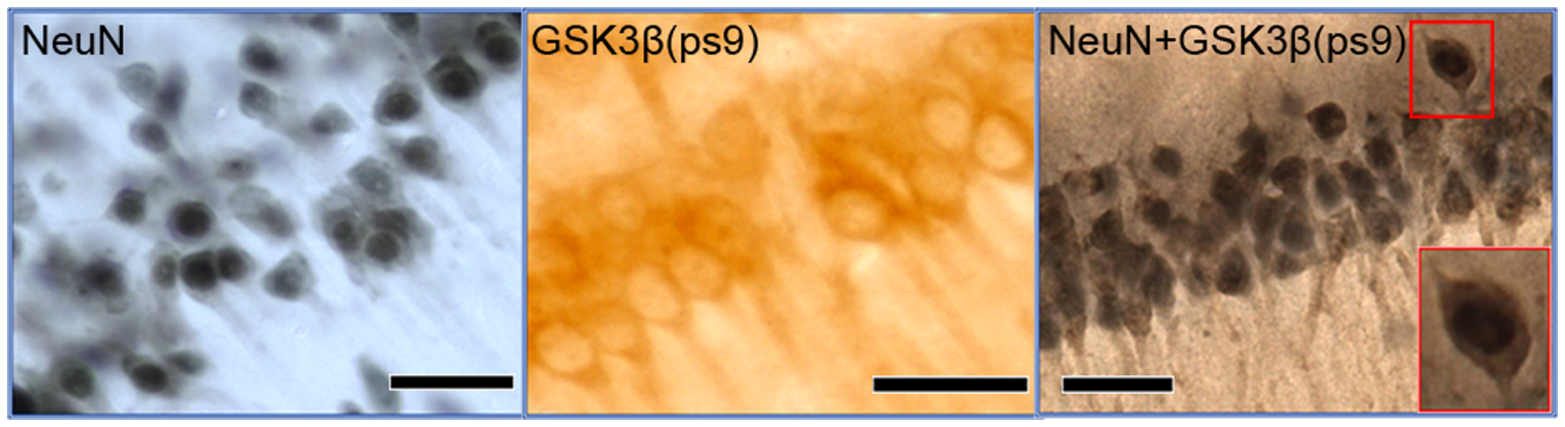
Immunopositive staining of CA1 pyramidal cell nuclei (NeuN) and inactive GSK3β (cytoplasm). To visualize pyramidal cells for cell counting an antibody against NeuN, a nuclear marker was used along with SG, a dark grey stain. Inactive GSK3β (GSK3βps9) in the cytoplasm of pyramidal cells in the CA1 was visualised using an antibody against GSK3βps9 along with DAB, a brown stain. A double immunopositive staining was performed to count the number of CA1 pyramidal cells that contained inactive GSK3β in the cytoplasm using antibodies against NeuN and GSK3βps9 with SG and DAB respectively. Scale Bar = 40 µm.

A 1-in-10 series of hippocampal sections (50 µm thickness) were taken and double immunohistochemically stained for NeuN, a neuronal marker (nuclear staining) and inactive GSK3β, phosphorylated at serine 9 (GSK3β_ps9_; cytoplasmic staining, figure 1). The tissue was washed in 0.1 M P.B, pH 7.4, and then subjected to a series of blocking steps using 1% w/v sodium borohydride (Sigma-Aldrich Company LTD, Gillingham, UK) in 0.1 M PB, pH 7.4, 10% v/v methanol (Fisher Scientific UK LTD, Loughborough, UK) and 3% v/v hydrogen peroxide (Sigma-Aldrich Company LTD, Gillingham, UK) in 0.1 M PB, pH 7.4 and 10% v/v BSA with 0.01% v/v Tween 20 (Sigma-Aldrich Company LTD, Gillingham, UK) in 0.01 M PBS, pH 7.4. This was followed by an overnight incubation in primary antibodies against NeuN (1:100, IgG monoclonal raised in mice; MAB377, Chemicon Europe Ltd., Chandlers Ford, UK) and against GSK3β_ps9_ (1:75, IgG polyclonal raised in rabbit; ab30619, Abcam, Cambridge, UK) diluted in 0.1% w/v BSA with 0.25% v/v Triton x100 (Sigma-Aldrich Company LTD, Gillingham, UK) in 0.1 M tris buffer saline (TBS, pH 7.6). After this the tissue was washed in 0.1 M TBS, pH 7.6 and incubated in the secondary antibody for NeuN (1:200 Biotinylated donkey IgG anti-mouse; 715-001-003, Jackson ImmunoResearch Europe Ltd, Newmarket, UK) diluted in 0.1% w/v BSA in 0.1 M TBS, pH 7.6 followed by a 0.1 M TBS, pH 7.6 wash and then incubated in an avidin DH and biotinylated horseradish peroxidise macromolecular complex solution (ABC solution; 2% v/v avidin and 2% v/v biotinylated enzyme in 0.01% v/v Tween 20 in 0.01 M PBS; Vector Elite ABC kit; Vector Laboratories LTD, Peterborough, UK) to localise and amplify the signal. After another wash with 0.1 M TBS, pH 7.6 an incubation in a Vector SG substrate kit, a grey-coloured chromagen (3% v/v SG chromagen and 3% v/v hydrogen peroxide in 0.1 M TBS, pH 7.6; Vector SG substrate kit for peroxidase, Vector Laboratories LTD, Peterborough, UK) was then used to visualise the NeuN peroxidase reaction. Following a wash in 0.1 M TBS, pH 7.6, the tissue was incubated in 20% v/v avidin D and 0.1% w/v BSA in 0.1 M TBS, pH 7.6, washed in 0.1 M TBS, pH 7.6 and then incubated in 20% biotin and 0.1% w/v BSA in 0.1 M TBS, pH 7.6 to block any remaining avidin-biotin binding steps (Avidin/Biotin blocking kit, Vector Laboratories, Peterborough, UK). The secondary antibody for GSK3β_ps9_ (1:200 biotinylated donkey IgG anti-rabbit; 711-001-003, Jackson ImmunoResearch Europe Ltd, Newmarket, UK) diluted in 0.1% w/v BSA in 0.1 M TBS, pH 7.6 was then used. Again this step was followed by washes and incubation in the ABC solution. The tissue was then washed in 0.1 M TBS, pH 7.6 and treated with 0.22% w/v 3,3′-diaminobenzidine (DAB; Fluka Chemie GmbH, Buchs, Switzerland) and 0.0001% v/v hydrogen peroxide in distilled water to visualise the peroxidase reaction. Following final washes with 0.1 M PB, pH 7.4 sections were then mounted on gelatin-coated glass slides, dehydrated through an ascending series of alcohols (30% to 100%), passed through xylene and glass cover slips were applied to the slides using Pertex. Controls for the immunohistochemical reactions were performed alongside the experiments.

### Cell Number and Shape

The pyramidal cells in the immunohistochemically-enhanced sections were visualised using a Nikon eclipse e80i microscope (Nikon UK LTD, Kingston-upon-Thames, UK) with an ultrafine 0.1 µm resolution motorised LEP x, y stage and motorised z axis at a magnification of x400 (Nikon Plan Fluor 40x/0.75 and optical lens CFI 10x/22) and the live image was relayed by a high resolution MicroFire™ 599808 digital camera (Optronics, Goleta, USA). Contours were drawn at low magnification (x40; Nikon Plan Fluor 4x/0.13 and optical lens CFI 10x/22) around the CA1 *stratum pyramidale* (SP) as defined by West et al. (1991) [46]. The stereologically unbiased method, optical fractionator, within the *StereoInvestigator*© version 7 software (MBF biosciences, Magdeburg, Germany) was used to count the pyramidal cells in these regions. The criteria for counting a ‘particle’, in this case the nucleus of a pyramidal cell, was a dark grey stained (NeuN-SG staining) nucleus with a faint outline of the entire pyramidal-shaped cell body, which was resident in the SP. If the particle was also to be marked positive for GSK3β_ps9_ staining it had to meet the pyramidal cell criteria and also have a brown cytoplasm (GSK3β_ps9_-DAB staining, figure 1). For a particle that fit the criteria to be ultimately counted it had to lie within a randomly placed counting frame such that it’s nuclear profile did not touch the red (forbidden) lines, however it may cross the green lines. A preliminary study for each cell count was performed with differing sizes of counting frame and grid to identify the ideal parameters for each test allowing the coefficient of error to be equal to or below 0.05 [47], [48]. The total number of cells counted (an average of 400 cells were counted per animal) was divided by the height sampling fraction (a height dissector of 20 µm divided by the mounted section thickness of 20 µm to 30 µm, which was measured during counting), the area sampling fraction (counting frame area of 1600 µm^2^ divided by the area of the sampling grid, which was 20000 µm^2^ for the total number of pyramidal cells in the CA1 SP and CA3 SP and 3600 µm^2^ for the number of pyramidal cells containing inactive GSK3β in the CA1 SP) and the section sampling fraction (1/10 as 1 in every 10 sections were used to perform the cell count).

The maximum and minimum diameters of CA1 pyramidal cell bodies were also calculated using *Neurolucida*© version 7 and *Neuroexplorer*© (MBF biosciences, Magdeburg, Germany). Three sections (anterior, medial and posterior dorsal hippocampal sections) previously used for the cell counts were inspected using the Nikon eclipse e80i microscope and the structures of interest were visualised at a magnification of 400X (Nikon Plan Fluor 40x/0.75 and optical lens CFI 10x/22). The structures were traced and the minimum and maximum diameters of the two types of structure were calculated. A preliminary ‘rolling average’ test was performed to establish the minimum number of structures traced that would give the lowest standard error of the mean (SEM) possible. The first point at which both average diameters (maximum and minimum) and their SEM became almost constant, even when the number measured was increasing, was taken as the ideal minimum. Twenty cell bodies per section were required for the CA1 pyramidal cell body measurements.

### Statistical Analysis

Statistical analysis was performed on all data using SPSS 16.0 for Windows (SPSS Inc., Chicago, USA). A one-sample t-test was performed on the average social recognition memory test results against the 0.5 ‘no recognition memory’ ratio. A one-way ANOVA followed by a post-hoc Tukey’s test was used to assess any significant differences between groups for all other results. The level of statistical significance was taken as P<0.05.

## Results

Neither FGL nor Aβ_25–35_ had an effect on the average body weight during the course of s.c. treatments (data not shown); the data correlate well with that of Cambon et al. (2004) [31], Borcel et al. (2008) [25] and Secher et al. (2006) [32]. These three studies found that FGL had no effect on body weight in rat pups using intranasal administration [32], and adult rats using i.c.v. [31] or s.c. administration [25], [32].

### Social Recognition Memory

**Figure 2:**
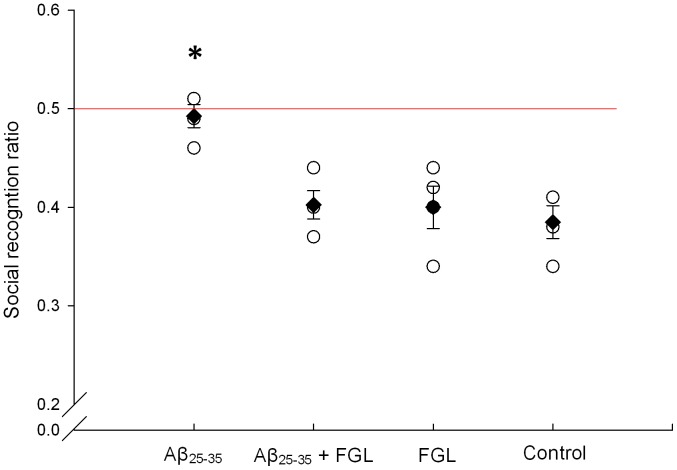
Average social recognition ratio on day 24. An unfamiliar juvenile rat was introduced into the cage of the test rat and 30 minutes later the juvenile was placed back in the cage. Both investigation times were recorded and the social recognition ratio was calculated. A social recognition ratio of 0.5 indicates no memory of the juvenile. Using a one sample t-test, it was found that animals treated with Aβ_25–35_ followed by FGL, FGL alone or vehicles (control) had significantly lower ratios than 0.5 (P<0.01), whilst animals given only Aβ_25–35_ did not. A one-way ANOVA was also performed on the individual social recognition ratios and animals given Aβ_25–35_ alone had significantly greater social recognition ratios than that of any other group (*P<0.05). The mean ratio for each group is signified by a black diamond (± SEM, n = 4), whilst the open circles indicate individual ratios in that given group.

The average social recognition ratio (SRR) for rats treated with Aβ_25–35_ only was not significantly different from the 0.5 ratio whilst the SRR of the Aβ_25–35_+FGL, vehicle+FGL and the control (vehicle+vehicle) groups were significantly lower than 0.5 and the Aβ_25–35_ alone group (figure 2; P>0.05, n = 4). This indicates that the Aβ_25–35_ only group was unable to recognize the juvenile rat during the second encounter, whilst the other groups did recognize the juvenile. This suggests that Aβ_25–35_ causes impaired short-term memory; however, when given FGL memory is rescued.

### Hippocampal Volume

**Figure 3:**
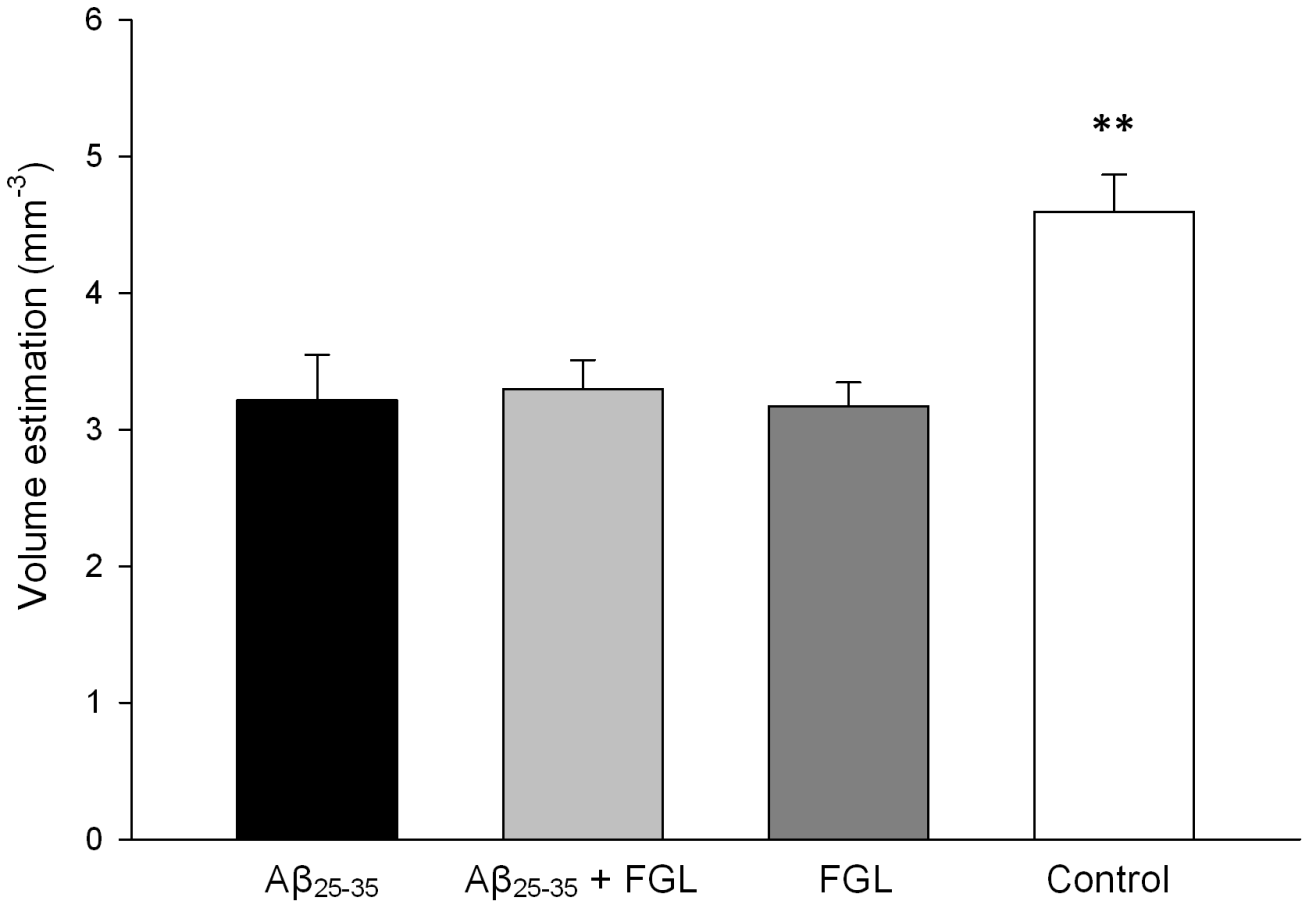
Volume estimations of the right dorsal CA3. Using a one-way ANOVA and Tukey’s post-hoc test, the volume of the dorsal CA3 in the control group was significantly larger than all the other groups (**P<0.01). Mean ± SEM, n = 4.

For the right hemisphere, there were no significant differences between the average volumes, measured for any of the groups, of the dorsal and ventral hippocampi, or the CA1, CA2, and the dentate gyrus (P>0.05, n = 4). There was a trend towards a reduction in volume of the right hippocampus, particularly in the CA1 and its subregions, in the groups given Aβ_25–35_ and surprisingly also when animals were treated with FGL alone. The volume of the dorsal CA3 region of the right hippocampus in control animals was significantly greater by 30% than in all the other groups (figure 3; P<0.05, n = 4) but this was not found in the ventral hippocampus. In the left hippocampus, the volumes were not significantly different to the right hippocampal volumes (P>0.05, n = 4). This shows that the i.c.v. injection and the systemic treatment had an equal bilateral effect on the volume of the hippocampi, and so the latter part of the study was performed on the right dorsal hippocampus only.

### CA1 Pyramidal Cell Morphology

**Figure 4:**
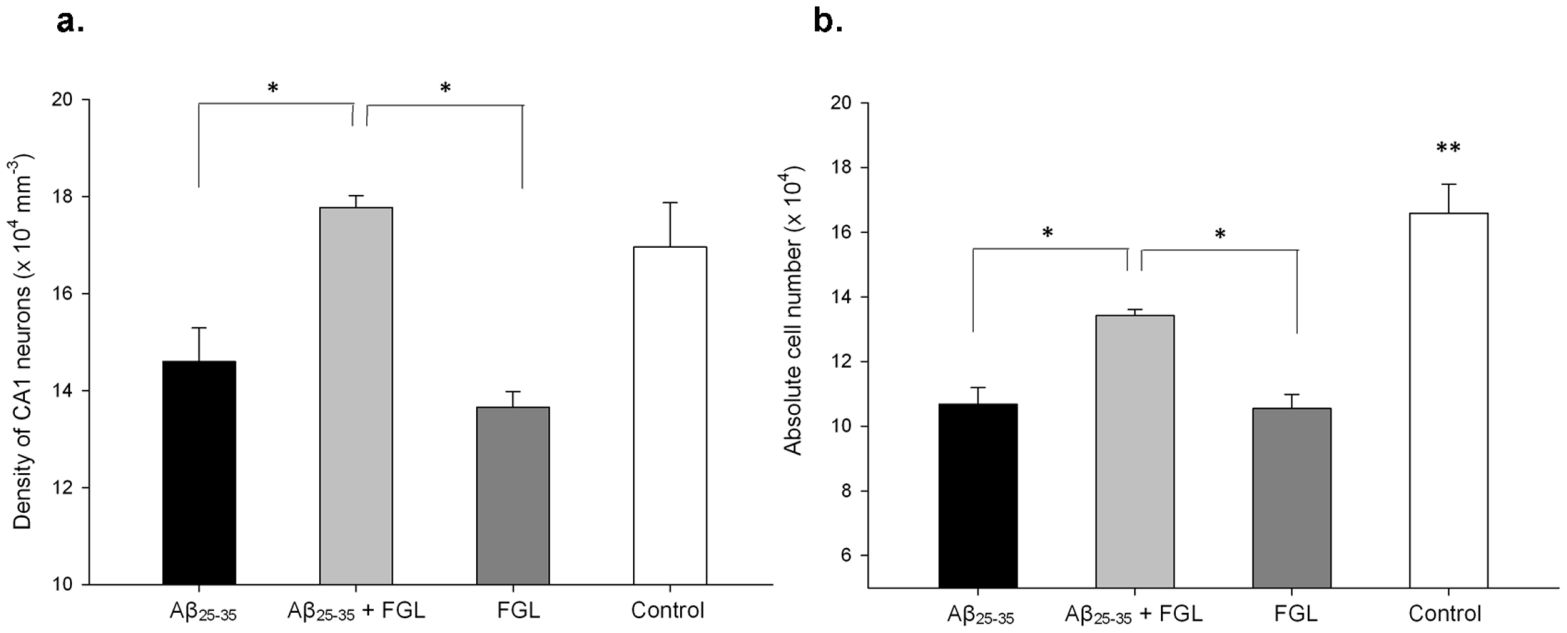
The density and total number of CA1 pyramidal cells in the right dorsal hippocampus. Immunocytochemistry (NeuN antibody in conjunction with DAB) and the optical fractionator method were used to establish cell density within the CA1. Cell density (a) was multiplied by the volume of the dorsal CA1 SP to establish total number (b). The data was analysed using a one-way ANOVA and Tukey’s post-hoc test. The Aβ_25–35_+FGL group had a significantly greater density (a) and total number (b) of pyramidal cells in the CA1 SP than compared with the Aβ_25–35_ alone and the FGL alone groups, whilst the control group had significantly more pyramidal cells than all of the other groups, regardless of the groups cell density. (*P<0.05, **P<0.01). Mean ± SEM, n = 4.

To investigate whether FGL could prevent Aβ-induced pyramidal cell loss in the CA1, a stereologically unbiased method, the optical fractionator [46] was used on 1-in-10 coronal immunocytochemically stained sections (using a NeuN antibody in conjunction with Vector SG substrate kit) throughout the right hippocampus to obtain pyramidal cell densities and absolute numbers in the CA1 SP (Figure 1). The average pyramidal cell density in the dorsal CA1 of Aβ_25–35_ alone rats was significantly lower than that of the Aβ_25–35_+FGL group (14% lower, figure 4a; P<0.05, n = 4). This was reflected in a significantly greater absolute number of pyramidal cells with Aβ_25–35_+FGL rats (by 22%) than Aβ_25–35_ alone rats (Figure 4b). The density of pyramidal cells in the CA1 of control rats was not significantly different to any of the other groups but the absolute number was significantly greater by 20 to 40% (P<0.05, n = 4). However, unexpectedly, animals given FGL alone had a significantly lower average density and absolute number of CA1 pyramidal cells compared to the Aβ_25–35_+FGL group and control animals (P<0.05, n = 4; Figure 4). These findings suggest that FGL alleviated Aβ-induced loss and density of CA1 pyramidal cells. However, FGL alone induced a significant loss of CA1 pyramidal cells.

**Figure 5:**
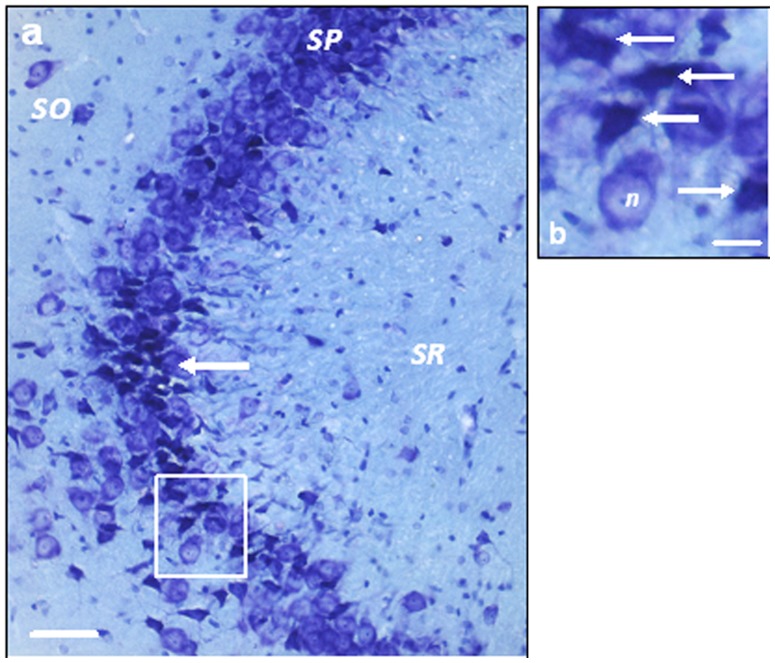
'Damaged' pyramidal cells in the right CA3. a) A section of the CA3 from a rat given Aβ_25–35_ alone, showing a large number of damaged pyramidal cells (arrow indicates a group of damaged pyramidal cells). b) An enlarged image of the box in a. The arrows indicate ‘damaged’ pyramidal cells with densely toluidine blue stained, concaved cell bodies. ‘n’ is an example of a ‘healthy’ neuron. Scale bar a = 50 µm and b = 20 µm.

In the CA3, there were no significant differences between any of the groups for either density of CA3 pyramidal cells or absolute number (P>0.05, n = 4). However, when qualitative observations were performed, ‘damaged’ pyramidal cells identified by the concaved shape and dense toluidine blue staining of the soma were found, particularly in the region that closely borders the lateral ventricle (Figure 5). The damaged cells were particularly prevalent in the groups treated with Aβ_25–35_ or FGL alone. The group given FGL following Aβ_25–35_, although still having a noticeable amount of damaged cells in the CA3 region, appeared to have a much reduced number of those cells compared to those groups. Damaged neurons were rarely seen in the CA1. This suggests that both Aβ_25–35_ and FGL had an effect on CA1 and CA3 pyramidal cells in the dorsal hippocampus.

To determine changes in shape and size of the cell somata of the dorsal CA1 pyramidal cells, 20 cells per section were traced at the maximum ‘in focus’ diameter. There were no significant differences between any of the groups regarding the maximum and minimum diameter. A ratio of the maximum and minimum diameter can represent the shape of the structure. A ratio of 1.0 signifies a spherical structure and a ratio less than 1.0 indicates the object has a prolate spheroid shape [49]. The ratio did not significantly differ between groups. The ratio ranged between 0.67 to 0.69 (P<0.05, n = 4) suggesting that the cells are still pyramidal in shape.

### Inactive GSK3β-containing CA1 Pyramidal Cells

**Figure 6:**
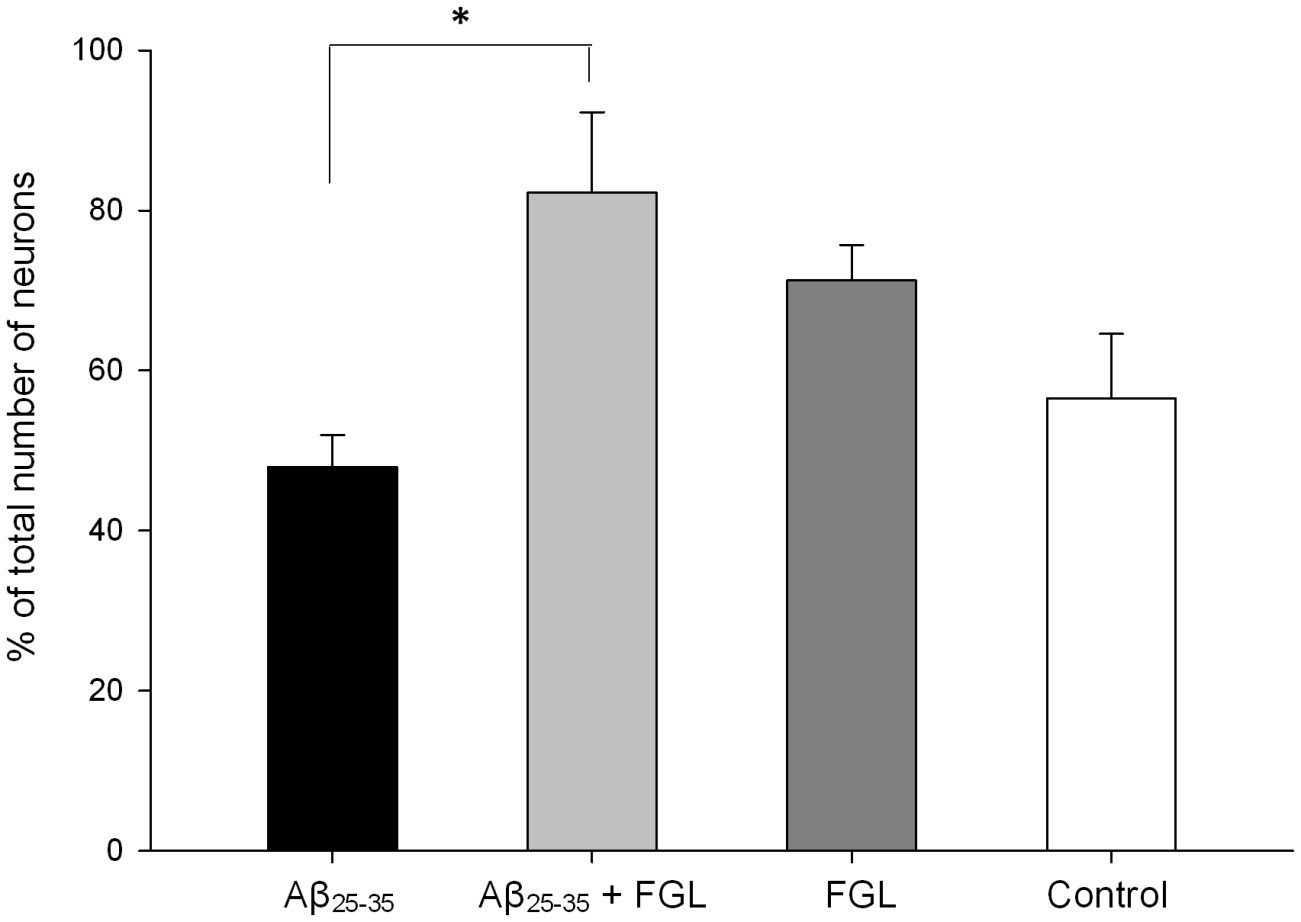
Percentage of CA1 pyramidal cells in the right dorsal hippocampus containing inactive GSK3β. Double immunocytochemistry and the optical fractionator method were used to establish the pyramidal cell density in the CA1 and also the density of pyramidal cells containing inactive GSK3β. The absolute numbers of both densities were calculated and the percentage of all the CA1 pyramidal cells that contained inactive GSK3β was established. The data was analysed using a one-way ANOVA and Tukey’s post-hoc test. Aβ_25–35_+FGL rats had a significantly higher percentage of pyramidal neurons in the CA1 that contained inactive GSK3β compared with Aβ_25–35_ alone rats. (*P<0.05). Mean ± SEM, n = 4.

The proportion of pyramidal cells containing inactive GSK3β was calculated to determine the effects of Aβ_25–35_ and FGL on the activation state of GSK3β, since both have been linked to this kinase. Prior to analysis several sections through the hippocampi of the animals were immunolablled for GSK3β (all forms), all pyramidal cells in the CA1 contained GSK3β regardless of treatment (data not shown). The percentage of inactive GSK3β-containing CA1 pyramidal cells was calculated using immunocytochemistry (figure 1) and the optical fractionator method. All FGL treated animals had an increased percentage of inactive GSK3β-containing CA1 pyramidal cells in the right dorsal hippocampus, with a significantly greater increase seen in those animals also treated with Aβ_25–35_, compared with the control animals and animals given Aβ_25–35_ alone (figure 6; P<0.05, n = 4), suggesting that FGL acts on GSK3β and has inactivated Aβ-induced increases in GSK3β levels.

## Discussion

Klementiev et al. (2007) performed preliminary work using three s.c. injections of 8.0 mg/kg FGL on this Aβ_25–35_ model and the authors suggested that FGL could be a potential treatment in AD [21]. The current study aimed to further progress the work using seven s.c. injections of 10.8 mg/kg FGL on the i.c.v. injected Aβ_25–35_ young adult rat, specifically in the right dorsal hippocampus. Although average hippocampal volume did not decrease significantly with Aβ25–35 administration there was cell loss and memory impairment 4 weeks later. FGL was found to alleviate Aβ25–35- short-term memory impairment, and an in-depth, detailed morphometric analysis using unbiased stereological techniques also showed alleviation of CA1 pyramidal cell loss. However, FGL administered to healthy rats caused detrimental effects, including neuronal damage and loss. FGL may conceivably exert its effects via the FGFR-Akt-GSK3β pathway.

### FGL Rescued Aβ_25–35_-induced Social Recognition Memory Impairment

Social recognition memory impairment is a key early stage diagnostic symptom of AD. At day 24 the average recognition ratio of rats given Aβ25–35 alone was not significantly lower than the ‘no recognition’ ratio of 0.5. This implies that the animals in this group were unable to recognize the juvenile rat during the second introduction suggesting that Aβ25–35 impaired short-term recognition memory. This finding is similar to that of Klementiev et al. (2007) who, using the same AD model, found that the rat’s recognition ratio was not significantly lower than 0.5 as early as 2 weeks after the administration of Aβ25–35 and this was maintained until the end of the study at 7 weeks [21]. Other studies using Aβ25–35 *in vivo* have shown that this fragment not only impairs hippocampal-dependent short-term memory but also spatial, working and long-term memory [36], [40], [41], [50]. When rats were given systemic treatment with FGLL (s.c. 10.8 mg/kg) after i.c.v. injection of Aβ25–35 they had a similar ratio to that of the control group, which was significantly lower than 0.5, and that of the Aβ25–35 alone group. This suggests that these animals were able to recognize the juvenile rat on the second introduction, similar to the control animals but in contrast with the animals given Aβ25–35 alone. This is also in agreement with the findings of Klementiev et al. (2007), who report a lower recognition ratio after three different routes of administration – suboccipital intracisternal 1.2 µg/µl, intranasal 8 µg/µl or s.c. 8 mg/kg injections – of FGLL on days 7, 10 and 13 of a 4 week study (day 0 - i.c.v. injection of Aβ25–35) [21]. Taken together these results suggest that, after a range of different administration routes, treatment durations and concentrations, FGL is able to prevent early Aβ25–35-induced short-term hippocampal memory deficits. FGL has been shown to be effective at preventing memory impairment in other conditions and diseases also [25], [51].

When FGL was administered to rats without Aβ_25–35_, the animals were able to recognize the juvenile animal quickly during the second exposure, and hence had a lower recognition ratio than 0.5 similar to the control group. This suggests that their memory was not improved with FGL, contrasting with the work of Secher et al. (2006) [32]. Secher et al. (2006), using the same memory test, found that two s.c. injections of 8 mg/kg FGL given to healthy rats one hour and 73 hours before the behavioural test, improved both short- and long-term social memory [32]. These contrasting results may be due to the differences in the treatment course, amount of FGL administered or the time when FGL was administered in relation to the time of the test. Regarding the latter, in the current study FGL was administered 2 days before the behavioural test and then a day after the test, whilst Secher et al. (2006) administered FGL one hour before the test [32]. Unlike the current study, FGL was present in the brain during memory consolidation. FGL may be reinforcing synapses, because *in vitro* studies have shown that FGL is able to causes a short-term facilitation of transmitter release, a long-term increase of synaptic efficacy, and enhance pre-synaptic function and synapse formation in the hippocampus [28], [31].

### FGL Alleviated CA1 Cell Loss and CA3 Cell Damage caused by Aβ_25–35_

Administration of Aβ_25–35_ resulted in a trend towards a reduction in the volume of the dorsal hippocampus, particularly in the CA1 and a significant decrease in the volume of the CA3 region. This could be due to the animals being sacrificed only 4 weeks after the Aβ_25–35_ injection, because Klementiev et al. (2007) found that i.c.v. injection of Aβ_25–35_ causes a significant decrease in total hippocampal volume at week 8, but no volume changes at week 4 [21]. This contrasts with human AD studies, where hippocampal volume has been found to be reduced even in the early stages of familial AD [52] and may be correlated with spatial memory impairments [15]. Stoub et al. (2006) report a decrease in hippocampal volume using MRI which was correlated to reduced declarative memory in amnesic mild cognitive impairment patients [53]. These patients are at high risk of developing AD, which suggests the atrophy contributes to declarative memory decline before AD has been diagnosed. These conflicting data may be attributed to species differences - rats versus humans. Rodent memory may be more susceptible to ultrastructural changes than human memory [54]. This is particularly evident in the present study as cell death occurred without any marked volume change suggesting that hippocampal volume may not be a reliable marker of disease progression in Aβ-treated rodents. The memory deficits seen in the Aβ_25–35_-treated rats may be due to the substantial loss of CA1 pyramidal cells. This was greater than previously reported by Klementiev et al. (2007), who observed only a 20% reduction at 4 weeks in the same AD model [21]. A 40% reduction was reported by those authors at 8 weeks correlating to hippocampal volume decreases. However, the authors did not segregate the pyramidal cell counts into CA1, CA2 and CA3, whereas in the current study there are differences in absolute pyramidal cell number between hippocampal subregions; the CA1 showed cell loss, whereas the CA3 did not. A range of human AD studies found a marked cell loss in the CA1 (40–60% decrease; [16], [19], [55]–[57]), and of interest is that Hyman et al. (1984) and West et al. (1994) found no loss in other subfields of the hippocampus of AD patients [16], [57]. This degree of cell loss is similar to that seen in the rat model used in the present study; however, it is important to note that the human CA1 pyramidal cell layer is approximately six times thicker than the cell layer in the rat [58], consequently a much greater number of CA1 pyramidal cells are lost in human AD cases.

When FGL was given to Aβ-treated rats the density of CA1 pyramidal cells was similar to that seen in the control animals and the total number of neurons was reduced by 20%; not by 40% as seen in Aβ_25–35_ alone treated rats. This suggests that FGL was able to partially prevent Aβ_25–35_-induced pyramidal cell death in the CA1. This is similar to findings by Neiiendam et al. (2004) *in vitro* [24] who found that 50 µg/ml of FGL (24 hour incubation) was able to prevent primary rat hippocampal neuron death after 20 µM Aβ_25–35_ incubation [24].

When FGL was administered alone it caused a large reduction in both the density and the absolute number of CA1 pyramidal cells in the hippocampus. This was also seen by Ojo et al. (2013) in 4 month old healthy rats given 10 systemic doses of 8 mg/kg FGL (s.c.) [59], but is in contrast with work by Popov et al. (2008), who found no volume changes in the dorsal hippocampus of aged (24 months old) rats treated with 8 mg/kg FGL (s.c.) [27]. The differences may relate to the use of aged animals in the study by Popov et al. (2008) [27] whilst in the current study and in the study by Ojo et al. (2013) [59] only young adult rats were used. Aging has detrimental effects, to a lesser extent than AD; for example, increased glial cell number and a decrease in absolute number of pyramidal cells [26], [54]. It might be speculated that in the study by Popov et al. (2008) [27] FGL is working to provide protection from the effects of aging. In the current study, the reduction in hippocampal volume but maintenance of memory in rats treated with FGL alone is similar to that seen in the active Aβ vaccine study, AN1792, which caused improved cognition and decreased amyloid plaque-load but decreased brain volume in human AD cases [52]. The yearly rate of volume loss in the hippocampus was greater in those patients given the vaccine compared with patients given a placebo. Fox et al. (2005) suggest that this could be due to the vaccine accelerating neuronal loss, clearing of Aβ, reduction in water content or decreased glial volume and number [52]. The right dorsal CA3 volume was reduced by all treatments; Aβ_25–35_ alone, FGL alone and administration of both Aβ_25–35_ and FGL, which may be a result of up to a 5-fold increase in number of damaged pyramidal cells in the dorsal CA3 of these animals (cell damage in Aβ+Veh>in Veh+FGL>in Aβ+FGL animals). The Aβ_25–35_ findings are similar to those of Arancibia et al. (2008), who found that i.c.v. injection of Aβ_25–35_ caused damaged pyramidal cells in the dorsal hippocampus as early as 4 weeks after Aβ_25–35_ injection [34]. The FGL alone findings are similar to the cell damage and loss, and volume decreases seen in the study by Ojo et al. (2013) [59]. This suggests that both Aβ_25–35_ and FGL, when given separately, cause CA3 pyramidal cell damage but when combined are less detrimental.

Together these results lend support to the idea that the effects of both FGL and Aβ_25–35_ are not CA1 specific as they both have effects on the CA3; however, they exert a greater effect on the CA1 than the CA3 as only cell damage occurs in the CA3 whilst complete cell loss occurs in the CA1 when both are given alone. The Aβ_25–35_ results are in agreement with Stepanichev et al. (2006), who noted that i.c.v. administration of Aβ_25–35_ has the greatest effect on the CA1 of the hippocampus [36], and with West et al. (2000), who found that the CA1 is the most vulnerable region of the hippocampus to neuronal loss (up to 58% loss) in AD patients [19].

### FGL Treatment Increases the Proportion of CA1 Pyramidal Cells that Contain Inactive GSK3β

FGL is thought to work via the FGFR-AkT pathway [24] leading to the inactivation of GSK3β [21]. Active GSK3β has been shown to cause a range of AD pathologies. In human AD studies upregulation of the GSK3 gene has been seen in the hippocampus [60]. Aβ_25–35_ is known to increase levels of active GSK3β in hippocampal neurons [38], [61]. In the current study, there were more CA1 pyramidal neurons containing inactive GSK3β in the animals given FGL. This suggests that FGL inhibited GSK3β activity. The current findings also show that the most significant amount of inactive GSK3β containing CA1 pyramidal cells were seen in those animals given FGL after Aβ_25–35_ administration. This could be a result of Aβ_25–35_-induced upregulation of GSK3β expression in the neurons, and hence FGL is able to inactivate a greater amount of GSK3β.

GSK3β is a regulator of apoptosis [62]. For the current study, the loss of neurons by Aβ_25–35_ alone could be a result of disregulation of GSK3β with Aβ_25–35_ promoting apoptosis as Hu et al. (2009) found that when Aβ oligomers were given to rats, there was an increased level of caspase 3 and TUNEL (a DNA fragmentation marker) staining in the CA1, suggesting that apoptosis had occurred [63]. FGL is thought to act as a GSK3β inhibitor opposing the effects of Aβ_25–35_, and hence regulating the kinase and prevent apoptosis. Rockenstein et al. (2007) have shown that administrating lithium (a GSK3β inhibitor) to human APP transgenic mice can alleviate memory deficits, protect dendritic structures, and reduced tau and APP phosphorylation in the hippocampus of those mice [64]. This pathway seems to be the most obvious link between FGL and Aβ, and how FGL can be a neuroprotective agent; however, it is important to establish the exact pathway of interaction via biochemical analysis.

The effect of FGL on GSK3β may also explain the detrimental effects seen in the healthy young adult rat hippocampus. Hu et al. (2009) reported very similar findings with a GSK3 inhibitor, SB216763 (SB), using *in vitro* and *in vivo* AD models [63]. SB was found to protect primary rat hippocampal neurons *in vitro* from Aβ oligomer toxicity; however, administration of a high concentration of SB alone caused toxicity. *In vivo* Aβ oligomers administered to rats were shown to cause increased activity of GSK3β, in the CA1, whilst SB effectively reduced but did not abolish GSK3 activity [63], correlating with the partial prevention mentioned in the present study. Similar to the current study, Hu et al. (2009) also report damaged neurons and dystrophic neurites in the CA1, CA3 and DG with Aβ treatment, which was prevented by SB; whilst SB alone caused damage to the hippocampal neurons [63].

The effects seen with FGL and SB could be due to their ability to inactivate GSK3β. Active GSK3β promotes mitochondria-mediated apoptosis (‘intrinsic’ apoptosis), whilst inactive GSK3β promotes death domain-containing receptor-mediated apoptosis (‘extrinsic’ apoptosis; as reviewed by [62]). In both forms of apoptosis GSK3β is thought to be upstream to the caspase signalling [62]. GSK3β upregulates the expression of transcription and translation factors, and proteins that are important in the apoptotic pathway and downregulates anti-apoptotic proteins, lowering the threshold for apoptosis. In a healthy cell, regulation of GSK3β inhibits either form of apoptosis from occurring [62]. GSK3β knockout mice die due to liver damage caused by hepatocyte apoptosis, whilst overexpression of GSK3β alone induces apoptosis of the pheochromocytoma-derived PC12 cell line [65].

For the current study, the loss of neurons caused by administration of FGL alone could be a result of disregulation of GSK3β leading to extrinsic apoptosis. For example, Song et al. (2004) were the first to show that lithium and other GSK inhibitors are able to potentiate extrinsic apoptosis in Jurkat cells and rat hippocampal neurons via the inhibition of GSK3 [66]. The loss of neurons may not occur in animals treated with Aβ_25–35_ and FGL because the opposing effects of Aβ_25–35_ and FGL on GSK3β may regulate the kinase and prevent apoptosis. For example, overexpression of GSK3β in the mouse forebrain (Tet/GSK3β mice) caused increased tau phosphorylation, neuronal apoptosis, reactive astrocytes and learning deficits. The pathologies were reversed and the levels of GSK3β were reduced after 6 weeks of the GSK inhibitor, doxycycline or silencing of the gene [67]. Gomez-Sintes et al. (2007) also found that dominant-negative (DN) GSK3 expressing mice (Tet/DN-GSK-3) had impaired motor coordination and increased levels of neuronal apoptosis, which were reversible if the DN-GSK3 expression was shut down [68]. Both Gomez-Sintes et al. (2007) and Rockenstein et al. (2007) have shown that DN-GSK3 expression or administration of lithium (respectively) to human APP transgenic mice can alleviate memory deficits, protect dendritic structures and reduced tau and APP phosphorylation in the hippocampus of those mice [64], [68]. The current hypothesis can also be supported by Hu et al. (2009), who found that when Aβ or SB were given alone that apoptosis had occurred in the CA1. However, when Aβ and SB were administered together apoptosis was inhibited as there was little or no staining for caspase 3 or TUNEL [63].

This study has demonstrated that i.c.v. injection of Aβ_25–35_ is detrimental to the CA1 and CA3 pyramidal cells, similar to that in human AD brains, potentially leading to short-term memory impairment. The administration of an NCAM-derived peptide, FGL, alleviated this pathology and memory impairment. However, FGL administered to healthy animals can be detrimental to the hippocampus. The effects of Aβ_25–35_ and FGL may be linked via GSK3β, allowing FGL to be beneficial in pathological conditions but detrimental to the healthy hippocampus.
